# Foodborne Diseases Due to Underestimated Hazard of Joint Mycotoxin Exposure at Low Levels and Possible Risk Assessment

**DOI:** 10.3390/toxins15070464

**Published:** 2023-07-19

**Authors:** Stoycho D. Stoev

**Affiliations:** Department of General and Clinical Pathology, Faculty of Veterinary Medicine, Trakia University, Students Campus, 6000 Stara Zagora, Bulgaria; s_stoev@hotmail.com

**Keywords:** feed safety, health hazard, risk assessment, hygiene control, prophylactic measures, mycotoxin regulations, mycotoxin interaction, foodborne diseases

## Abstract

The subject of this review paper is to evaluate the underestimated hazard of multiple mycotoxin exposure of animals/humans for the appearance of foodborne ailments and diseases. The significance of joint mycotoxin interaction in the development of foodborne diseases is discussed, and appropriate conclusions are made. The importance of low feed/food levels of some target mycotoxins co-contaminations in food and feedstuffs for induction of target foodborne mycotoxicoses is also studied in the available literature. The appropriate hygiene control and the necessary risk assessment in regard to possible hazards for animals and humans are also discussed, and appropriate suggestions are made. Some internationally recognized prophylactic measures, management of the risk, and the necessity of elaboration of new international regulations in regard to the maximum permitted levels are also carefully discussed and analysed in the cases of multiple mycotoxin contaminations. The necessity of harmonization of mycotoxin regulations and control measures at international levels is also discussed in order to facilitate food trade between the countries and to ensure global food safety.

## 1. Introduction

Mycotoxins are natural contaminants and are ubiquitously present in various commodities of plant origin, mainly in cereals, but also in oilseeds, nuts, vegetables, cocoa and coffee beans, fruits, dried fruits, wine, beer, and spices. In addition, contamination with mycotoxins is reported in animal products such as meat, eggs, milk, and milk derivatives, if the same animals are exposed to mycotoxin-contaminated feed [1,2,3,4,5]. It is well known that most mycotoxins represent a significant risk to the feed or food supply and have a great impact on international trade and the economies of affected countries. It is obvious that a high percentage of feed or food samples have been found to contain more than one mycotoxin [2,3]. Often, the established contamination levels were low enough to comply with European requirements and guidance values and were below the maximum permitted levels. Unfortunately, mycotoxin co-contamination, which is often found in the same feeds and foods, might still exert adverse effects on animals or humans because of additive or synergistic interactions between some of the mycotoxins [4,5,6,7]. On the other hand, mycotoxins significantly impact both the productivity and the nutritional value or quality of cereal and feedstuffs. The most important challenge of food safety worldwide is to ensure healthy feeds or foods free from dangerous contamination levels of any single mycotoxin and to protect humans and animals from food-born ailments [3,7]. Nowadays, the situation regarding global food security is still worrying, and food safety issues have been found to increase over the last decades. Therefore, the questions about the effectiveness of current regulatory measures and control systems are still of great importance because mycotoxins are one of the most important contributing factors to the condemnation of foods/feeds or food loss, especially in developing countries. Mycotoxin contamination in such countries not only decreases feed/food quality but also decreases export values and leads to significant economic losses [7].

Nowadays, over 400 types of different mycotoxins have been identified, but only 10–15 are really important for public health, e.g., aflatoxins (AFs) with aflatoxin B1 (AFB1) and aflatoxin M1 (AFM1) being the most important, deoxynivalenol (DON), ergot alkaloids, fumonisins (FUMs) with fumonisin B1 (FB1) being the most important, ochratoxin A (OTA), patulin (PAT), zearalenone (ZEA), and trichothecenes (mainly T-2 and HT-2) because the same are often found in animal feed or human food and have harmful effects on both animals and humans [2,3]. Contamination of feeds or foods may occur both during pre-harvest and post-harvest. Direct consequences of exposure to mycotoxins contaminated feed include different common problems in farm animals, such as decreased feed intake, feed refusal, poor feed conversion, diminished body weight gain, increased disease incidence, and reduced reproductive capacities [4]. According to their biological activities, mycotoxins may have carcinogenic (e.g., AFB1, OTA, and FB1), oestrogenic (ZEA), neurotoxic (FB1), nephrotoxic (OTA and FB1), immunosuppressive (AFB1, OTA, and T-2 toxin) or genotoxic (AFB1, OTA, T-2) effects [5].

The World Health Organisation (WHO) and the Codex Alimentarius Commission (CAC) have recently developed some new limits for food safety in the international food trade [6]. In this regard, the European Community has also introduced maximum permissible limits for most mycotoxins [7]. Unfortunately, these regulations do not address various mycotoxin combinations, which often have strong synergistic effects in significantly lower contamination levels than permitted ones. For example, such a strong synergistic interaction is seen between OTA and penicillic acid (PA), mycotoxins produced by the same ochratoxinogenic fungi responsible for such contamination in the field [8,9].

In order to ensure appropriate food safety control and to prevent foodborne diseases, target quality control measures have to be introduced, which should be part of a well-designed and coordinated international system. In this regard, the knowledge of qualified professionals in targeted scientific fields, such as food science and technology, veterinary medicine, health science, and agriculture, is crucial for the implementation of these safety and quality control activities. However, only an integrated approach and introducing the Hazard Analysis and Critical Control Point (HACCP) system could help resolve the existing problems and improve food safety management via systematic identification and hazard assessment in food production and establishing subsequent target measures for appropriate control.

The objectives of this review paper are to evaluate the underestimated hazard of multiple mycotoxin exposure of animals/humans for the appearance of foodborne ailments and to elucidate the significance of joint mycotoxin interaction in the development of foodborne diseases, which are supported by some appropriate pictures. Another important objective is to suggest appropriate hygiene control and the necessary risk assessment in regard to possible hazards for animals and humans in some target mycotoxicoses. The objective of the review is also to elucidate the suitable management of the risk and the necessity of elaboration on new international regulations in regard to the maximum permitted levels in the cases of multiple mycotoxins contaminations and to recommend the necessity of harmonization of mycotoxin regulations and control measures at international levels.

## 2. Underestimated Hazard of Joint Mycotoxin Exposure and Possible Foodborne Ailments

### 2.1. The Cause and Modes of Joint Mycotoxin Exposure of Animals/Humans

**Figure 1:**
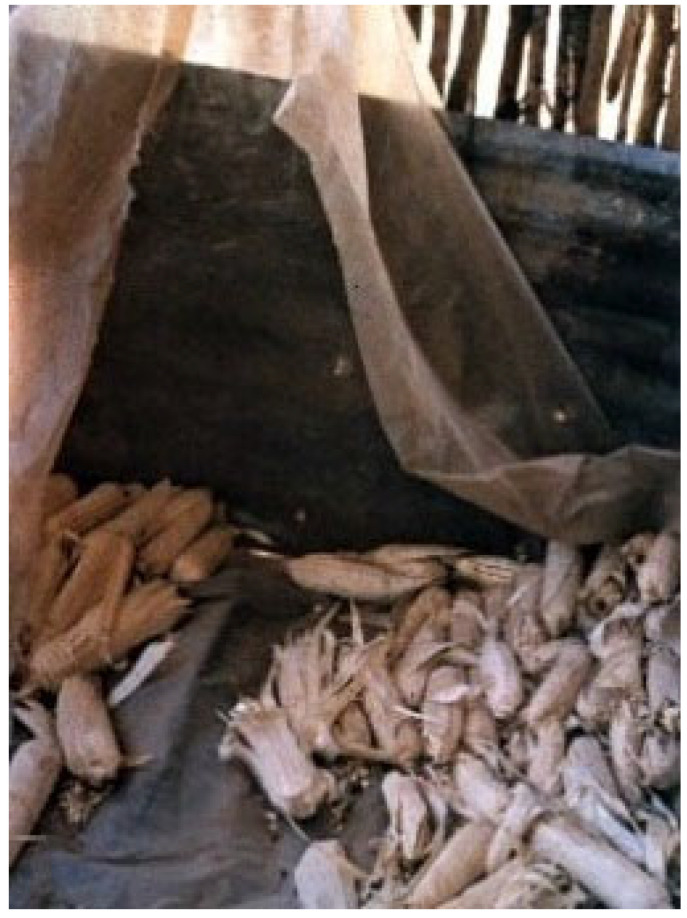
An inappropriate store in rural area of Limpopo province of South Africa, which would not be able to preserve feed from rain [7].

**Figure 2:**
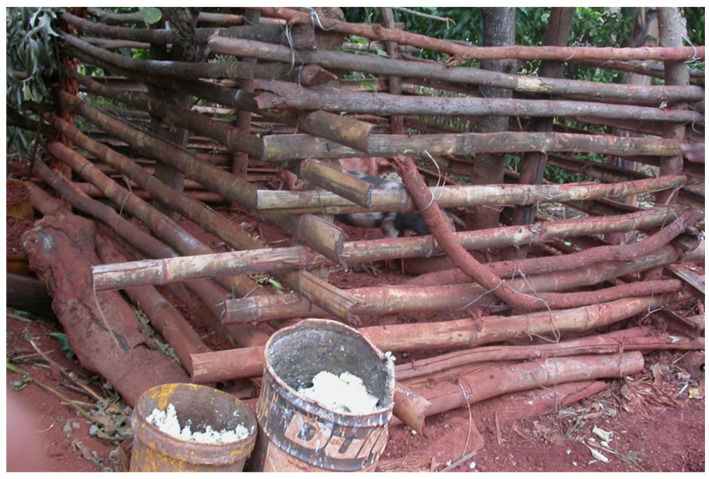
An inappropriate manner for keeping the feed for pigs in rural area of Limpopo province of South Africa; the feed is exposed to atmospheric influence and is often additionally moulded [7].

**Figure 3:**
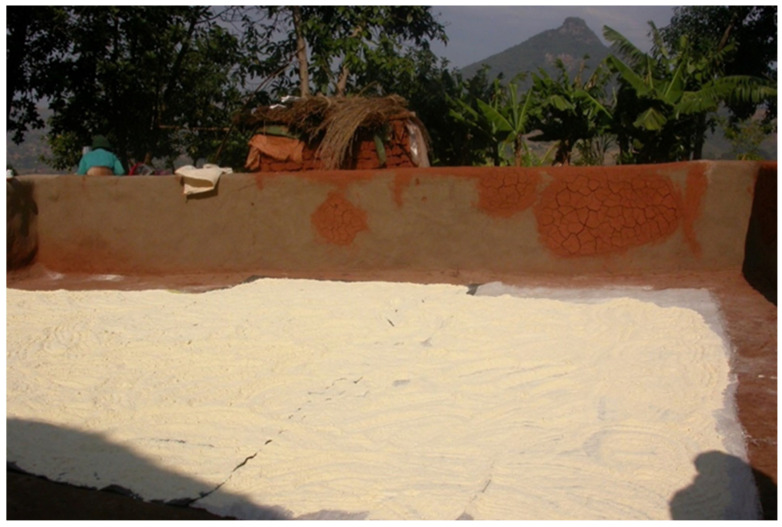
An inappropriate manner for drying or preserving maize flour in a rural area of Limpopo province of South Africa [7].

Despite the extensive efforts to control fungal contamination of feed or food, mycotoxin co-contamination has often been reported in cereals, feedstuffs, and food commodities, which represents a great concern due to the observed negative health effects on animals and humans. The survey on the fate of mycotoxins during cereal processing, such as milling, revealed that mycotoxins are concentrated into fractions that are often used as animal feed [7]. In addition, because of the possible carry-over of mycotoxins, feed contamination can also contribute to the increased contamination of food products from animal origin and mycotoxin exposure in humans and subsequent health burden [1,10]. Therefore, mycotoxin contamination of food or fodder is a serious problem for humans and animals in many areas of the world [11,12,13,14,15,16,17], mostly in developing countries, and such contamination often provokes some foodborne ailments or diseases [7]. Mycotoxin production is often unavoidable depending on certain environmental conditions in the field during the growth of target plants susceptible to mould infestation or during the storage of food and fodder, e.g., temperature stress and high moisture content (water activity) (Figure 1, Figure 2 and Figure 3). The invasion of cereals by fungi occurs in both cases, after the harvest or in the field. Therefore, the potential for multi-toxin contamination is high. Some mycotoxins are produced by fungi in growing cereals prior to harvesting, and therefore, any control would be difficult to perform. However, mycotoxin production during storage is much easier to prevent by drying the cereals after collecting the crops, which usually suppresses the growth of fungal species from *Penicillium* or *Aspergillus* genera [18]. The poor storage practice and inappropriate manner of keeping the animal feed exposed to atmospheric influence may often result in additional mycotoxin contamination and fungal growth (Figure 1 and Figure 2) [19]. Additionally, mycotoxin contamination is often reported in vegetables; fruits; and animal products such as meat, milk, dairy products, and eggs [17,20], which additionally increases animal/human exposure to some target mycotoxins.

It is important to emphasize that most mycotoxins are thermally and chemically stable and, therefore, persist during the storage and most of the production- or thermal processes [21]. The foods/fees are the main source of human or animal mycotoxin exposure, whereas ingestion is the main route of mycotoxin intake. Nevertheless, mycotoxin inhalation can also contribute to mycotoxin intake in people exposed to organic dust during the storage work, milling process, etc., because the fungal spores or hyphae fragments of fungi in the airborne dust usually serve as carriers of mycotoxins [22,23].

### 2.2. Foodborne Ailments and Underestimated Hazard from Joint Mycotoxin Exposure

**Figure 4:**
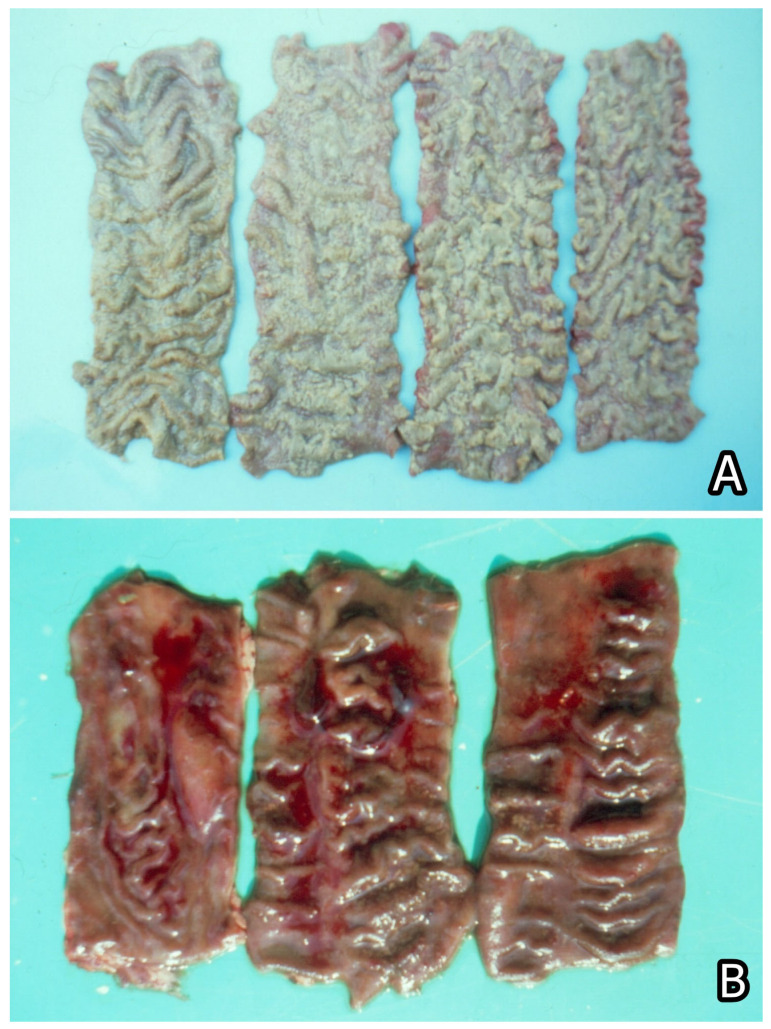
(**A**) Diphtheroid accretions on colon mucosa of a pig given 3 ppm ochratoxin A for 17-days and sick by secondary salmonellosis. (**B**) Haemorrhagic dysentery associated with *Serpulina hyodysenteriae* and *Campylobacter coli* in pigs immunised against *S. choleraesuis* 47 days after commencing a diet containing 1 ppm ochratoxin A.

**Figure 5:**
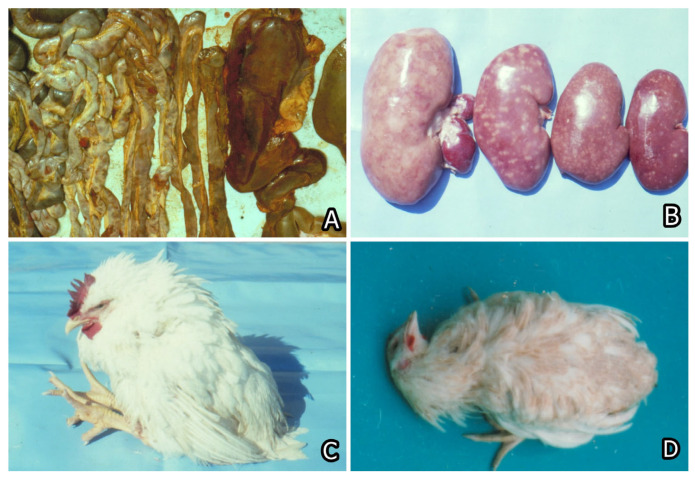
(**A**) Spontaneous case of Stachybotryotoxicosis in cattle. Strong haemorrhagic diathesis on the serosa of the intestine and ulcerative-necrotic damages, which are translucent under the serosa. (**B**) Macroscopic appearance of kidneys with mycotoxic nephropathy. Different degrees of enlargement and mottled or enlarged and pale appearance of kidneys in pigs of 6–8 month-age from Bulgaria. Enlargement of renal lymph nodes. (**C**) Nervous symptoms such as flexion of legs and sitting posture in a chick fed on mouldy diet containing 790 ppb OTA and 2000–5000 ppb PA for 10 months. (**D**) Nervous symptoms such as torticollis in a chick fed on mouldy diet containing 790 ppb OTA and 2000–5000 ppb PA for 70 days [7].

The multiple mycotoxin contamination of foods and fodder has been reported frequently and was considered responsible for many ailments in animals or humans [7,24,25,26]. The toxic effects of mycotoxins or mycotoxin combinations and their importance for the development of various human and animal ailments or livestock diseases are well known. Some secondary bacterial infections attributable to mycotoxins have been reported (Figure 4), as well as some fusariotoxicoses such as alimentary toxic aleukia in humans, equine leukoencephalomalacia and porcine pulmonary oedema, and vulvovaginitis and rectal prolapse in pigs or human oesophageal carcinoma. The animal ailments known as aflatoxicoses, stachybotryotoxicosis (Figure 5A), mycotoxic porcine/chicken nephropathy (Figure 5B), ergotism, and many others are also well known [7,17,27,28].

The moulded maize was known in the past (more than 160 years ago) to be responsible for the death of horses and pigs, which was then found to be due to the toxic fungi, mainly *Fusarium moniliforme (F. verticillioides)*. Unfortunately, the FUMs, the mycotoxins responsible for these mycotoxicoses, were not known until the 1980s, when the same were discovered [29,30]. FUMs, however, can also be produced by *A. niger* and, therefore, could not be considered *Fusarium* mycotoxins [31]. The first hazard assessment of these mycotoxins, e.g., FB1, was evaluated just after some outbreaks of equine leukoencephalomalacia [32,33] and porcine pulmonary oedema in the USA between 1989 and 1990, which were responsible for the death of many horses and pigs, fed with mouldy maize contaminated with FUMs [34]. It has been established that the observed damage in the brain of horses and in the lungs of pigs is a consequence of targeted disorders in vessel function and increased permeability of endothelial cells provoked by impaired sphingolipid metabolism [35]. In addition, FB1 was found to provoke a disturbance in myocardial contractility in pigs, which is possibly provoked by the inhibition of L-type calcium channels in the myocardium by increased sphingosine [36]. Therefore, FB1 could additionally complicate pulmonary oedema in pigs by provoking heart failure. Such a disturbance in myocardial contractility in humans was first established in 1980 in rural hospitals in South Africa. The same was called Idiopathic Congestive Cardiopathy (ICC)—heart weakness due to the increased influx of blood in the hearth. The sick patients were usually elderly people, fed on a diet containing home-produced maize and drinking a lot of homemade beer. This circumstance suggests that a possible cause of the same cardiopathy could be FB1, together with other *Fusarium* mycotoxins [7,28]. FB1 also causes some other negative effects on humans and animals, e.g., teratogenic, hepatotoxic, and nephrotoxic, and was also suspected to induce esophageal cancer, liver cancer, and neural tube defects in humans [37]. A high content of this mycotoxin has been found in foods intended for the human diet but also in milk, meat, and eggs [3].

ZEA is another Fusarium mycotoxin, often found in mouldy multi-mycotoxin contaminated maize, which exerts oestrogenic activity in animals. Cold, wet periods and the early onset of frost, followed by periods of sunshine, facilitate the infestation of crops with *Fusarium* spp. The same mycotoxin and the other *Fusarium* mycotoxins are mainly produced before the harvest [38]. It is mainly reported to contaminate maize, barley, oats, or wheat and is often found in cereal products such as bread, pasta, and beer [39] but is also seen in milk, meat, internal organs, and eggs [3]. ZEA was found to be responsible for vaginal or rectal prolapse in swine, vulvovaginitis, swelling of the mammary glands, infertility, and some other estrogenic symptoms [7,38,40,41,42]. Among its estrogenic effects, the most pronounced include increased embryo lethal resorptions, decreased fertility, reduced litter size, change in serum levels of progesterone, and teratogenic effects in pigs and sheep [43]. At high doses, ZEA can disrupt ovulation, implantation, fetal development, and the viability of newborn animals [44]. Increased exposure to ZEA is associated with abortions in dairy cattle, reduced feed intake, poor reproductive performance, decreased milk production, vaginitis, and mammary gland enlargement in heifers. Female pigs were found to be the most sensitive to ZEA, having some clinical symptoms such as swelling of the vulva and mammary glands, prolonged estrus intervals, vulvovaginitis, stillbirth, vaginal and/or rectal prolapse, ovarian atrophy, abortion, and infertility [7,45]. In male pigs, ZEA provokes feminization and a decrease in spermatogenesis, testicular weight, libido, and testosterone levels [44]. ZEA is also a toxin that could provoke vomiting, oxidative stress, nausea, or diarrhea, in addition to its estrogen activity, which may lead to human female reproductive changes or cervical cancer and could be partly responsible for premature enlargement or breast in puberty [46,47]. It also has a genotoxic and cytotoxic effect and strong embryological toxicity and could be partly responsible for esophageal cancer or breast cancer [47,48,49]. ZEA was also seen to be carcinogenic in mice and to induce hepatocellular adenomas and pituitary tumours [50,51]. However, more epidemiological studies with reliable exposure assessment are necessary to confirm or reject its possible carcinogenicity in humans.

The hyperestrogenism in female pigs (vulvovaginitis or rectal and vaginal prolapses), which was thought to be provoked by the high feed levels of ZEA, occurred only after prolonged exposure to the same mycotoxin (around a month), whereas the first clinical symptoms of exposure to feed contaminated with *Fusarium graminearum* and/or *Fusarium culmorum* are usually connected with some target toxic effects of DON, e.g., cytotoxic (degenerative changes in internal organs and gastrointestinal tract), emetic (gastrointestinal damages), neurotropic (paresis of the limbs), and immunosuppressive (secondary bacterial infections) effect [7,28]. Female pigs are the most vulnerable animals among all farm species to DON toxicity. The main symptoms provoked by DON in pigs are vomiting (DON is known as “vomitoxin”), feed refusal, and skin hemorrhage. DON can also reduce milk production in dairy cattle, whereas low levels of exposure to DON can induce nausea, diarrhea, lesions in the gastrointestinal tract, growth retardation, damage in the haematopoietic systems, and immune dysregulation [52,53,54]. Poultry was found to be less sensitive to DON, and feed refusal is only observed at very high contamination levels in feedstuffs (16–20 mg/kg feed) [55]. Among animals, ruminants were found to be the least sensitive animal species to DON, which is due to the capability of rumen microflora to detoxify it [56]. This mycotoxin has been mainly reported to contaminate wheat, corn, rye, oats, barley, and rarely rice, triticale, or sorghum. Cereals are usually contaminated by DON in the field, but also at storage time [3].

Fusariotoxicosis provoked by *Fusarium* spp. belonging to the section *Sporotrichiella* or *F. poae* species and less often involving the *F. tricinctum* species was thought to be due mainly to mycotoxins T-2 toxin, HT-2 toxin, and diacetoxyscirpenol (DAS), which have a strong cytotoxic, immunosuppressive, and genotoxic effect in animals and humans, and after prolonged exposure, accumulate in various tissues in the body [7]. T-2 and HT-2 toxins are known as two of the most toxic mycotoxins among the type A trichothecenes. Oats and oat products were reported to be particularly susceptible to contamination with high levels of T-2 and HT-2, followed by barley [11]. T-2 toxin can inhibit protein synthesis, subsequently leading to some side effects of DNA and RNA synthesis and to a decrease in cellular immune response [57]. T-2 can also induce destruction in the haematopoietic system, decreased egg production, damaged eggshells, growth retardation, and feed refusal [58]. In addition to T-2 and HT-2, DAS also exerts acute and chronic effects, such as hematotoxicity, growth retardation, lung disorders, and cardiovascular effects, which could be additive or synergistic to these ones provoked by T-2 and HT-2 [7]. Pigs and poultry are the most sensitive species, whereas ruminants are the less sensitive because of the protection of microflora in the rumen [11].

The abovementioned fusariotoxicosis provoked by *Fusarium* spp. belonging to the section *Sporotrichiella* or *F. poae* species is seen after exposure to a diet containing a high quantity of grain and hay or straw, wintering in the open. In humans, this intoxication is known as alimentary toxic aleukia [59]. Toxins, responsible for this fusariotoxicosis, possess a strong irritative action and provoke catarrhal haemorrhagic gastroenteritis necrosis; ulceration in the digestive tract; degenerative changes in the kidney, liver, heart, and brain; and peripheral ganglia of the vegetative nervous system, which subsequently leads to some spasms or tremors in the muscles or paresis of the hind limbs. Some damaging effects are also found in the wall of blood vessels, which subsequently cause a haemorrhagic diathesis [7]. Symptoms of acute intoxication include nausea, tremors, abdominal pain, diarrhea, and weight loss [60].

Another problematic mycotoxicosis is known as ergotism and is provoked by the fungus *Claviceps purpurea,* which produces a lot of mycotoxins, e.g., lysergin amide derivatives such as ergine and ergometrine; peptide alkaloids such as ergosine, ergotamine, and ergosecaline; biogene amines such as histamine and acetylcholine; ergocristine, ergocryptine, and some others. These mycotoxins are found in the sclerotia of this fungus, which actually present a cemented compact mass of hyphae, very similar to a large and dark wheat grain. Among the abovementioned mycotoxins, the most important for ergotism are ergometrine, ergotamine, and ergocristine [7,38]. The most often affected cereals include rye, barley, wheat, oats, and millet. Rye is the cereal where this fungus forms sclerotia (dark crescent-shaped bodies). This mycotoxicosis, also known as St Anthony’s fire, has been reported to cause hallucination, and it has been responsible for the death of many people in European countries, especially France, during the Middle Ages. It is also one of the oldest food-borne diseases known in humans [61]. The main chronic symptoms of the disease are the gangrene of peripheral parts of the extremities and subsequent loss of the same peripheral parts of the limbs as well as some gastrointestinal ailments [62,63]. Horses and ruminants are among the most sensitive animals to this mycotoxicosis. The mycotoxins involved in the ergotism also provoke an increased contractility of the uterus and many subsequent abortions or prolapse of the uterus. The main cause for ischemic necroses in peripheral body parts, e.g., extremities, tail, ears, crown of hooves, etc., is considered the strong vasoconstriction of blood vessels [62,64].

Stachybotryotoxicosis is another important mycotoxicosis that is seen often in farm animals. It is provoked by the fungus *Stachybotrys alternans (Stachybotrys altra),* which often contaminates the moist substrates (hay/straw/oats) containing a large quantity of cellulose. Contaminated hay or straw is recognized by its black coating formed by the fungus. Mycotoxins responsible for this mycotoxicosis are satratoxins, verrucarins, and roridins. These mycotoxins initially irritate the mucosa of the gastrointestinal tract, provoking hyperaemia in the mouth and nasal areas and swelling of the lips and fissures of the mouth. The same mycotoxins cumulate in the tissues of animals provoking damage in the vessels, which are responsible for haemorrhages and oedematous changes. Some damages were also seen in the haematopoietic tissue, which subsequently led to leukopenia and thrombocytopenia. Degenerative changes in all internal organs and secondary (neurotrophic) deep symmetrical areactive necroses and ulcers in the mouth and digestive tract are also characteristic features of this mycotoxicosis [7,28,65,66] (Figure 5A). The fact that this fungus can survive for a long time outside the substrate, e.g., in the soil, is a very disturbing circumstance, which leads to the repeated contamination of feeds and explains the stationarity of stachybotryotoxicosis.

AFs are among the mycotoxins which were discovered due to their strong toxicity and devastating effect on turkey poults or other poultry in 1960 in the UK when above 100,000 turkey poults were dead as a result of aflatoxicosis [67]. The target organ affected by AFs appeared to be the liver, which was usually yellow and enlarged. The same disease, previously known as “Turkey X disease”, was found to be due to high contamination levels of AFs in Brazilian peanut meal, which has been imported as a feed ingredient. Feed or food contamination with AFs was found to be most often and in high contamination levels in countries from Africa, Asia, and South America possessing warm and humid climates. Such contamination, albeit at low levels, is also seen in temperate areas of North America and Europe. AFs are reported to mainly contaminate maize, peanuts, pistachios, and cotton seeds, but their levels increase significantly during storage [3]. The most sensitive are young animals and ducks or turkeys. The characteristic features include fatty degeneration and necroses on the liver, haemorrhages, enlargement of the gall bladder, oedema of the wall of the gall bladder and yellowish tissue staining around it, proliferation in the interstitial tissue of the liver, and gastrointestinal damage. In addition to liver damage, mild degenerative changes were also seen in other organs. In chronic intoxication, the main symptoms are usually hydrothorax, ascites, icterus, and cirrhosis of the liver, whereas, in the cattle, a characteristic thickening of the skin around the mouth and neck, as well as papillomas on the abomasal mucosa, were often seen [7,38]. In addition, the other chronic toxic effects of AFs include anemia, immunosuppression, and reduced growth rate [68]. The health and production of many animals, especially in poultry species, are strongly compromised by AFB1 exposure via the feed and can lead to growth retardation and serious economic losses [69]. The main clinical features of AFB1 toxicosis in humans include jaundice, diarrhea, ascites, abdominal pain, and vomiting [17]. The primary target organ in humans is, again, the liver, which could produce acute cirrhosis and necroses. AFB1, together with other mycotoxins, could induce edema in malnourished people and is also associated with Kwashiorkor disease [70,71]. AFB1 is also considered the most potent natural carcinogen and is classified by IARC as carcinogenic to humans (group 1) and causes up to 28% of all liver cancers [71]. AFs also have a teratogenic effect and can cause some deformities to the embryos by crossing the placental barrier [72].

Another important and dangerous mycotoxin is OTA, which was considered the main causative agent of mycotoxic porcine nephropathy (MPN) [73,74,75] and possibly involved together with some other toxins in Balkan endemic nephropathy in humans [26,76,77,78]. In addition to kidney damage, which is the major damage in pigs (Figure 5B) [24,25,74,79], low weight gain, some nervous signs (Figure 5C,D), liver changes, and a decreased weight of the eggs could also be seen in OTA-treated chicks or laying hens [80,81]. Some studies, however, revealed that in some countries, e.g., Bulgaria and South Africa, this nephropathy has more complicated etiology and pathology [78] attributed to the combined toxic effect of several mycotoxins such as OTA, PA, FB1, and not yet identified nephrotoxic metabolite (UM), having a proved synergistic interaction [24,25,82]. Some of these mycotoxins, such as OTA and PA, are usually produced under storage conditions, mainly in feeds/food prepared from fibrous plants or cereals, stored at high humidity for a long time. The other mycotoxin FB1, involved in this nephropathy, is usually produced in field conditions by fungi contaminating cereals (mostly maize) before harvesting [19]. The pig farms with a high incidence of such nephropathy are found to have various kinds of problems with the storage of the feed. In some cases, however, the problems were seen to come from certain feed plants using not properly dried grains collected in rainy or moist periods for preparing fodder. All farms which used such feed were subsequently reported to have nephropathy during slaughter, and their pigs were found to have decreased weight gain. The observed growth depression among such pigs, however, quickly disappeared after changing the feed source [74,83]. Such a growth depression and decrease in the weight of the eggs have also been reported in OTA-treated laying hens [80,81]. Ruminants are less sensitive to OTA because their rumen microflorae can degrade OTA to the less toxic OTα [84].

PAT is another mycotoxin having a variety of complex toxic effects, including acute toxicity, carcinogenicity, cytotoxicity, neurotoxicity, and reproductive toxicity [70,85,86], which is often found in multiple mycotoxin contamination. It usually provokes degenerative changes in the liver, kidney, and gastrointestinal tract but could also induce some additional disturbances in endocrine glands, the immune system, and other important organs and systems [87]. PAT has also been found to have a damaging effect on intestinal barrier function and to affect the normal intestinal flora [88,89]. The IARC defines PAT as a suspected carcinogen to humans, and therefore, it is classified as a Group 3 human and animal carcinogen [90,91]. A study performed in France revealed a significant exposure of pregnant women to PAT. It is also known that vegetarian mothers who ate more fruit per day were expected to be exposed to high levels of PAT [90,92]. The PAT often reveals its presence as a disease (rot) that affects apples after harvest or during storage. This mycotoxin has been reported in apples, apple juice, pears, grapes, and fodder affected by brown rot, but also in cereals and vegetables or different types of cheese [3]. If the consumers eliminate the rotten part of fruits before consumption or processing, the maximum permitted level for food safety is rarely exceeded. Heat processing manages to reduce the PAT content at a moderate level [3].

**Figure 6:**
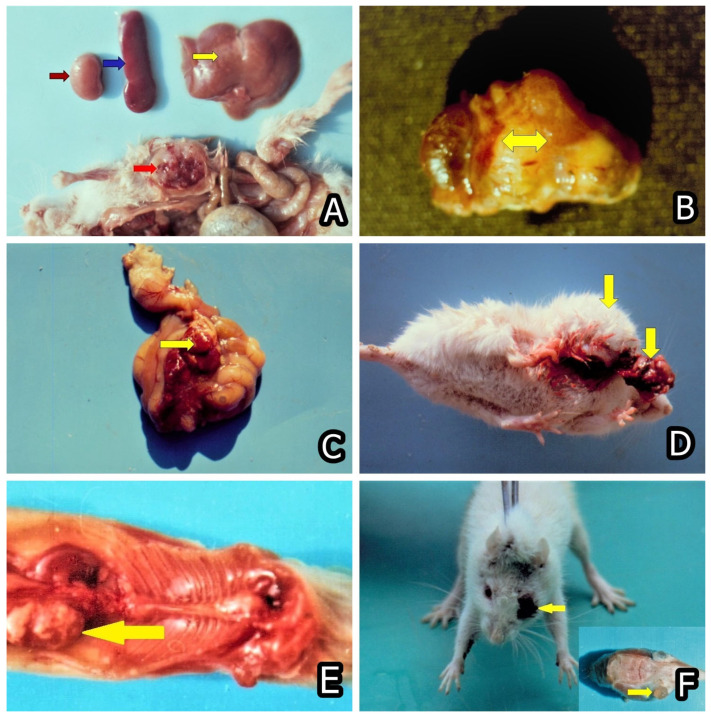
(**A**) Rhabdomyosarcoma in the muscles (red arrow), necroses in the liver (yellow arrow) and enlargement of the spleen (blue arrow), and pale color of kidney (brown arrow) in a mouse that died between months 15–20 and was exposed to 10 ppm OTA and 50–60 ppm PA. (**B**) Carcinoma in the region of kidney (yellow arrow) in a mouse that died between months 15–20 and was exposed to 10 ppm OTA and 50–60 ppm PA. (**C**) Angiosarcoma in the intestinal mesenterium (yellow arrow) in a mouse that died between months 15–20 and was exposed to 10 ppm OTA and 50–60 ppm PA. (**D**) Subcutaneous sarcoma (yellow arrows) in a mouse that died between months 15–20 and was ex-posed to 10 ppm OTA. (**E**) Adenocarcinoma in the kidney of rat exposed to 5 ppm OTA via the feed, which was slaughtered at the end of the 24th month of the experiment. Large grey-white neoplastic foci, which often merged with each other and protruded above kidney’s surface. (**F**) Squamous cell carcinoma in the eye (yellow arrow) of a rat exposed to 10 ppm OTA via the feed, which was slaughtered at the end of the 24th month of the experiment. The neoplasia spread over the eyelid, conjunctiva, and cornea [95,96].

**Figure 7:**
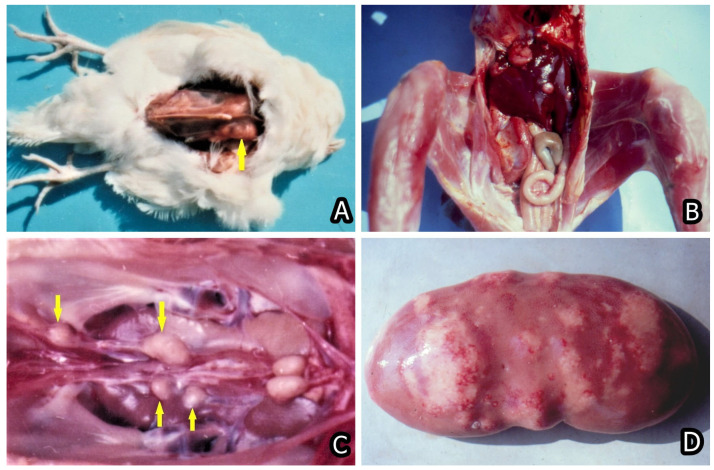
(**A**) Rabdomyoma in the breast muscle (yellow arrow) of female chick exposed to 5 ppm OTA and 25 ppm PHE via the feed, which was slaughtered at the end of the 24th month of the experiment. Large neoplasia in the region of breast muscle, which protruded significantly above the surface. (**B**) Adenocarcinoma in the liver of male chick exposed to 5 ppm OTA via the feed, which died at the end of the 10th month of the experiment. Large grey-white neo-plastic foci in the diaphragmatic surface of the liver protruded significantly above the surface. (**C**) Carcinoma in the region of ureters (yellow arrows) of male chick exposed to 5 ppm OTA via the feed, which died at the end of the 20th month of the experiment. Large grey-white neoplastic foci are seen along the ureters and protrude significantly above their surface. (**D**) Neoplastic tissue proliferation (fibroma and fibroadenoma) in kidney with spontaneous mycotoxic porcine nephropathy [74,94].

**Figure 8:**
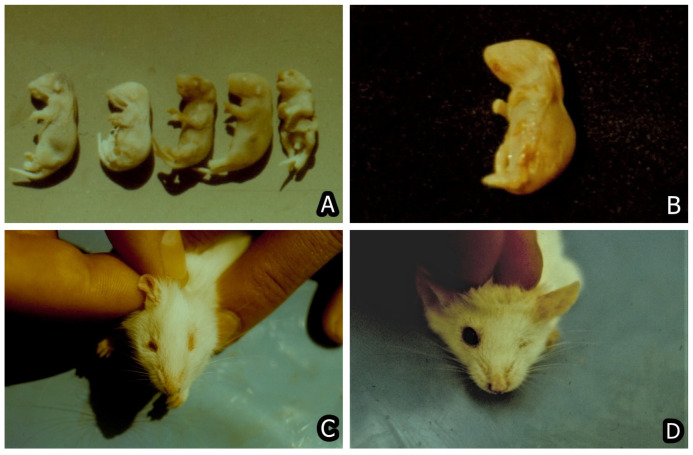
(**A**) Malformations in newborn mice whose mothers were exposed to 20 ppm OTA and 6 ppm OTB in the feed given from day 7 up to day 12 of the pregnancy—astomia and anophthalmia (both fetuses on left), 2 normal fetuses in the centre and spina bifida, e.g., facial cleft and maxillary hypoplasia (the fetus on right). (**B**) Malformations in newborn mice whose mothers were exposed to 20 ppm OTA and 6 ppm OTB in the feed given from day 7 up to day 12 of the pregnancy—peromelia in both right legs, astomia, and anophthalmia. (**C**) Malformation (micromonoftalmia) in a mouse whose mother was exposed to 10 ppm OTA via the feed up to day 8th of the pregnancy. (**D**) Malformation (monoftalmia) in a mouse whose mother was exposed to 10 ppm OTA via the feed up to day 8th of the pregnancy [98].

Nowadays, the main animal or human health problem in regard to mycotoxins intake is related to chronic effects, e.g., immunotoxic, carcinogenic, teratogenic, hepatotoxic, nephrotoxic, and hormonal/endocrine-disrupting effects. Therefore, the main concern in regard to most mycotoxins appeared to be their carcinogenic (Figure 6A–F and Figure 7A–D), genotoxic, immunotoxic, and teratogenic effects (Figure 8A–D), in addition to their acute toxic effects [47,93,94,95,96,97,98,99,100]. For example, FB1 was first discovered because it was involved in the induction of human esophageal cancer in South Africa [101]. The same mycotoxin was subsequently proved to have a carcinogenic effect on the rats’ liver [102], to be a nephrotoxin [103,104], and to be involved in human or animal nephropathies in Bulgaria and South Africa [24,25] together with OTA and PA [19].

Most of the investigations on the associations between mycotoxins intake and cancer risk addressed AFB1, whereas the other mycotoxins, e.g., DON, PAT, ZEA, and T-2 toxin [T-2], are classified as Group 3 mycotoxins by IARC because of the lack of enough data from both animal and human carcinogenicity. For FB1 and OTA (Group 2B), there is enough evidence of carcinogenicity in experimental animals (Figure 6A–F and Figure 7A–D) but insufficient data on humans [71]. Therefore, some additional animal and human studies need to be undertaken in order to clarify their involvement in cancer development. So far, there are a few studies addressing only the possible effects of chronic mycotoxin exposure on cancer risk, but the focus is on single mycotoxin exposure and in vitro studies [105]. However, there are scarce in vivo studies in farm animals in regard to the chronic effect of multiple mycotoxins exposure and the effect of such exposure on cancer development, as well as the possible synergistic or additive effects between mycotoxins [106,107,108].

It was presumed that various degenerative changes and neoplasia found in the liver, kidney, or intestinal parenchyma in OTA-treated mice/rats could be associated with the rout of OTA-elimination [94,95,96] (Figure 6A–F). It is well known that mice and rats excrete a very large proportion of OTA via the bile (around 33% hepatobiliary rout of excretion), which distinguishes the same species from pigs and humans, eliminating OTA mainly through the kidneys [109]. In this regard, neoplasia in the liver and gastrointestinal system, which represents a large proportion of OTA-induced tumours in rats [96], could be due to degenerative changes in the same organs [110] and enterohepatic recirculation of OTA in rats [111,112].

The other important and widely encountered mycotoxin AFB1 is not only a hepatotoxin [113] but also has carcinogenic, genotoxic, and immunotoxic effects on animals/humans, suppressing both cellular and humoral immune response and provoking growth retardation in animals [114,115,116]. Similarly, both mycotoxins OTA and FB1 have different deleterious effects on animals/humans, being simultaneously nephrotoxins [8,9,73,74,76,79,117], immunotoxins [118,119], genotoxins [99,114], with teratogens with craniofacial deformities being the most prevalent (Figure 8A–D) [98,120], and carcinogens [94,95,96,97,121,122] (Figure 6A–F and Figure 7A–D).

More attention should be paid to the strong immunosuppression induced by mycotoxins, which is usually the first observed toxic effect of mycotoxins and often clinically apparent before the characteristic clinical symptoms for each mycotoxicosis. For example, high susceptibility to natural infections and secondary bacterial diseases has been demonstrated in pigs exposed to OTA-contaminated diets at levels similar to those in practice [119] just after 1–2 weeks of OTA exposure, when the known kidney damage can not be recognized. Obviously, in this case, the humoral immune response was affected to the extent of allowing the development of secondary bacterial infections at a very low feed level of OTA of 1 ppm (Figure 4).

Another example of increased susceptibility to target microbial infection, e.g., *Mycobacterium bovis, Salmonella typhimurium*, *Staphylococcus,* and *Lysteria monocytogenes,* is reported in animals and chicks exposed to T-2 toxin [123,124,125,126,127]. A study also demonstrated significant interactions of T-2 toxin with *Salmonella typhimurium* pathogenesis, resulting in bacterial intoxication. In the same study, the T-2 toxin was found to decrease the amount of *Salmonella typhimurium* bacteria present in the cecum contents, but simultaneously, the susceptibility of porcine macrophages and intestinal epithelial cells to *Salmonella typhimurium* invasion was increased [123].

Some cases of increased mortality (13.2%) in *Salmonella typhimurium*-challenged broiler chicks [128], increased colonization of duodenal and caecal content of *Salmonella typhimurium* [129], increased damages in internal and immunocompetent organs in *Salmonella enterica*-challenged broiler chicks [130] are reported, when the same chicks were simultaneously exposed to OTA. The presence of OTA in poultry rations was also seen to increase mortality and the severity of an *E. coli* infection [131]. Some controversial results are reported in the development of some parasitic diseases, such as coccidiosis in OTA-exposed chicks. Huff and Ruff [132] reported a decreased severity of *Eimeria acervulina* and *Eimeria tenella*-induced coccidiosis when the chicks were simultaneously exposed to OTA. However, most of the other authors reported increased mortality in *Eimeria tenella*-induced coccidiosis in chicks [133] or more heavy progress and mortality of duodenal coccidiosis provoked by *E. acervulina*, when the same chicks were exposed to OTA as can be seen from the higher value of lesion and oocyst indices [134]. A similar increase in mortality or morbidity was also seen in experimental coccidiosis provoked by *Eimeria adenoeides* in turkey poults when the same poults were exposed to OTA [135].

Similarly, a heavier or more complicated progression of *Pasteurella multocida* infection [136] and porcine reproductive and respiratory syndrome (PRRS) [137,138,139] was seen in pigs exposed to FB1. According to Taranu and collaborators [140], the proinflammatory effect of FB1 is induced by the decreased phagocytic activity of pulmonary macrophages [136] and the subsequent increase in permeability of capillaries in the lung [117]. It is suggested that the immunosuppressive capacity of FB1 is due to the accumulation of free sphingoid bases in FB1-treated animals, which is responsible for the subsequent inhibition of lymphocyte proliferation [140]. In addition to the inhibition of cellular immune response, FB1 has also been reported to have a significant immunosuppressive effect on humoral immune response in pigs exposed to 10 ppm FB1 and immunized against Morbus Aujesky disease [117].

Therefore, the increased morbidity/mortality among chicks/animals fed on a diet containing target mycotoxins could be explained by the increased susceptibility to secondary bacterial disease [119] (Figure 4) and to the heavy progression of some widely spread parasitic diseases [133,134] or microbial/viral infections [117,118,119,123,137,138,139,141] due to suppressed humoral and cell-mediated immune responses.

Most mycotoxins can also induce oxidative stress [142,143] and, therefore, additionally damage animal/human health.

## 3. Appropriate Hygiene Control, Risk Assessment, and Possible Hazard for Animals and Humans

Food security is the base of human health and can ensure a high quality of life. It is well known that mycotoxin contamination of food/feed decreases their quality and export values. In regard to animal feed and human food, five to six mycotoxins (AFs, FUMs, DON, OTA, ZEA, and T-2) are of particular importance as the same are often contaminants in most agricultural commodities in the field and during storage. Therefore, the same mycotoxins are covered by EU legislation, e.g., EU regulation and recommendation. The EU has set the maximum permitted levels or guidance value for target mycotoxins in some animal feeds and human foods (Table 1 and Table 2) [144,145,146,147,148,149]. USDA (United States Department of Agriculture) also has set such limits in the USA (USDA, 2017) (Table 2). In addition to the regulated mycotoxins, several other non-regulated mycotoxins, e.g., emerging and modified mycotoxins, were also reported in some feed/food or foodstuffs and could represent an additional health burden [150,151,152]. Also, co-exposure to some target combinations of mycotoxins has also been commonly reported [7,24,25,38,153], and their combined synergistic or additive effects on animal/human health should be additionally considered and taken into account when defining the maximum permitted levels of the same mycotoxins [7,38,153]. 

Nowadays, the main scientific efforts are aimed at ensuring safe food/feed and preventing unsafe food/feed from being placed on the market via some appropriate systems for the identification of food safety problems and protection of human/animal health. The health-based cost-effectiveness analyses of two potential AFs control strategies in the African continent (such as pre-harvest biocontrol, using atoxigenic strains of *Aspergillus flavus* to competitively exclude toxigenic strains from colonizing maize and another similar strategy) revealed that such measures could be extremely cost-effective when applied widely. For example, the quality of life and health benefits gained from each strategy in terms of fewer aflatoxin-induced hepatocellular carcinoma (HCC) cases far exceeds the cost of control measures at the post-harvest time [154].

All these circumstances impose some additional measures to assess the current human/animal exposure to some target mycotoxins and/or mycotoxin combinations in Europe. Such effective monitoring could prevent future health impacts, especially in regard to chronic mycotoxin effects, e.g., immunotoxic, teratogenic, and carcinogenic effects [39]. Recently, the focus was set on DON and FB1, for which there are scarce data regarding human/animal exposure and subsequent hazard from such exposure [155].

It is well known that three major components of risk analysis, e.g., risk management, risk assessment, and risk communication, ensure target systematic measures for the protection of human/animal health [156]. It should be taken into account that the ‘risk assessment’ could be performed only via implementing the four major steps: hazard identification, hazard characterization, exposure assessment, and risk characterization. The exposure assessment step also includes the choice of exposure biomarkers and the analytical methods for the evaluation of the magnitude and frequency of mycotoxin exposure and the identification of the respective groups of animals/people that could be highly exposed [39]. The hazard assessment also requires the identification and evaluation of the main health effects of mycotoxins on animals or humans and, therefore, implies more experimental studies. On the other hand, ‘risk communication’ addresses the effective exchange of knowledge via the analysis of the respective risk. However, the “scientific risk assessment”, in some cases, could not ensure the complete information on which a “risk management decision” could be taken because some other factors should be taken into account, e.g., economic, societal, ethical or environmental factors as well as the control feasibility [156]. In this regard, the risk management of mycotoxin contamination in foods/feeds could be ensured only by the introduction of the HACCP approach (integrated system based on the Hazard Analysis and Critical Control Point), which should include target measures for control, prevention, good manufacturing practices, and quality control at all stages of agriculture production from the ripening of the crop up to the final consumer. This HACCP system is now applied to target mycotoxins, and a comprehensive manual is elaborated by FAO for the prevention and control of the same mycotoxins [157]. The same HACCP system is used for the identification of the major steps for preventing mycotoxin contamination of feeds/foods and the introduction of preventive strategy for targeting mycotoxins contamination in addition to possible measures for mycotoxins removal from the foods/feeds. In this regard, it is important to identify the stages at which effective monitoring can be realised. A good example of such an approach is the automatic sorting and segregation of peanuts for removal of the aflatoxin-contaminated nuts or target cleaning measures for cereals prior to milling, which facilitates the removal of the fungal spores and broken grains heavily contaminated with mycotoxins [157,158].

The strict enforcement of food safety laws and surveillance control is crucial for improving the security of food and preventing target food-borne diseases. However, such regulations and food safety laws must be regularly updated by introducing new scientific discoveries, e.g., the strong synergistic effect between OTA and PA (mycotoxins produced by the same fungi) [8,9,95], and new updated rules should be introduced in addition to the maximum permitted content for each single mycotoxin, addressing target mycotoxin combinations, which often have much stronger synergistic and carcinogenic effect in significantly lower contamination levels than permitted ones. In this regard, in addition to the regular updating of food safety laws via introducing new scientific achievements, a subsequent harmonization of different national standards and elaboration of common standards and regulations all over the world for each single mycotoxin or target mycotoxin combination in various raw materials or food commodities should be undertaken for improving the protection of consumers and the global food safety [7]. In such a way, in addition to food safety, fair international trade will be ensured. However, the international regulations and food safety laws should be carefully updated because the very restrictive laws will be followed by unjustified rejections of raw food materials or commodities, which could have some disastrous consequences for target producers, and some unjustified trade barriers could be created instead.

In other to ensure the increased safety of the food chain and compliance with EU legislation for food and feed, The Rapid Alert System for Food and Feed (RASFF) was created in 1979. RASFF serves as a useful tool for exchanging knowledge between competent authorities on consignments of food or feed in particular cases when a high risk for human/animal health has been identified and appropriate preventive measures have been undertaken [13].

The contamination of cereals with moulds and mycotoxins can be reduced via the introduction of some preventative measures before or after the harvest, e.g., timely harvesting, cleanup measures, sorting, drying of moistened grain, good storage practices, ozone fumigation, electromagnetic radiation treatment, chemical control agents, the introduction of corn resistant to fungal infestation, prevention of insect infestation, crop rotation, and others [3,7,159,160]. It is well known that failure to implement good agricultural practices, drought stress, or insect infestation are important factors that increase mycotoxin contamination [161]. Moreover, insufficient regulations and legislation can further contribute to the high incidence of mycotoxin contamination [162]. Good agricultural practices and storage protocols could also prevent or minimize mycotoxin contamination and should be introduced worldwide as much as possible. The complete removal of mycotoxins is usually hardly achievable in real practice, but the fate of each mycotoxin at each stage of feeds/foods production should be carefully examined in order to decrease the number of target mycotoxins in the food/feed products and to protect the final consumer when the risk assessment of the contaminant shows that its exposure level is unacceptable.

Many countries have established regulations and laws to provide food safety for their human or animal population. The first limits for mycotoxins were set for AFs in the 1960s. At the end of 2003, nearly 100 different countries, representing nearly 87% of the world population, set limits for mycotoxins in foodstuffs and feedstuffs, and the number continues to increase further [163]. The permitted maximum limits for mycotoxins were developed by various international or national organisations in the EU as well as in some other countries all over the world, e.g., Joint FAO/WHO Expert Committee on Food Additives (JECFA), World Health Organisation (WHO), and EU Scientific Committee for Food (SCF). Considering the increased information related to the changes in mycotoxins content at the time of food processing, more lenient limits for raw ingredients could be set if a significant decrease is seen in some stages of the food processing chain. Therefore, in-depth knowledge about how mycotoxins destroy or eliminate when using target techniques of foods/feed processing could further justify those foodstuffs for humans or animals that could contain high contamination levels of target mycotoxins. Such foodstuffs should be a subject of more frequent monitoring compared to the other products [164,165]. This knowledge is especially important for DON, ZEA, and FUMs in maize and cereals because the same mycotoxins are often found in very high levels in raw ingredients but are significantly lowered at the time of food processing. Such knowledge should be taken into account in order to ensure an appropriate balance between economic issues and the respective risk for the consumer because the absence of such a balance could lead to unnecessary penalisation of the agricultural producers without significantly improving food safety. It is important to emphasize that some food processing technics, such as bran removal, might significantly decrease human/animal mycotoxin intake but simultaneously might decrease the health benefits ensured by the final product. This issue raises the question of whether wholemeal bread, with the known benefits for health and possibly higher mycotoxin content, should be preferred compared to white bread, which usually has a lower mycotoxin content [166].

The European Commission (EC) accepted the maximum permitted limits for six groups of mycotoxins for animal feed: AFs, OTA, ZEA, FUMs, DON, and rye ergot, and seven groups for human food: AFs, OTA, ZEA, FUMs, DON, PAT, and T-2 + HT-2) and seven groups for human food: AFs, OTA, ZEA, FUMs, DON, PAT, and T-2 + HT-2) (Table 1 and Table 2). EU regulatory limits range from 0.1 μg/kg for AFB1 in processed cereal-based foods for human infants and young children to 4000 μg/kg for FB1 and FB2 in unprocessed maize for human consumption, whereas in regard to milk and milk-based products, the limit for AFM1 is 0.05 μg/kg. Maize is generally a good substrate for the production of mycotoxins, such as AFB1, OTA, ZEA, and DON, and, therefore, requires continuous monitoring. The contamination with the same mycotoxins represents a real health risk because of the high consumption of maize [3]. The most common mycotoxin in barley is DON, whereas the most common mycotoxins in cereal porridge are AFs and DON, and in breakfast cereals—AFs [3]. The mycotoxins that contaminate fruits and vegetables are mainly OTA, PAT, and trichothecenes [3]. The highest concentrations of OTA have been identified in southern Europe, and the same mycotoxin is mainly found in wine in the EU and USA but appears more often in red and sweet wines compared to white wine [167]. OTA is also the mycotoxin found most often and in high levels in coffee, cocoa, and chocolate [168,169,170]. On the other hand, DON is the most abundant mycotoxin in beer and is, therefore, the biggest public health problem related to beer consumption [171]. However, some other mycotoxins, such as OTA, AFs, ZEA, and FUMs, can also be found in the beer at different stages of brewing [171]. In peanuts and pistachios, the most common mycotoxins are AFs [3]. In addition to AFs, OTA is a significant mycotoxin that has been found in dried meat and, therefore, human exposure to this mycotoxin via meat or meat products, e.g., sausages and salamis, presents a global public health problem [172,173]. ZEA and DON were also found as meat contaminants but to a lesser degree [3]. On the other hand, the mycotoxins, known as the most significant contaminants of milk and dairy products such as cheese or yogurt, are AFs [3]. Regarding eggs, the most frequent contaminants are AFB1, OTA, ZEA, and DON [174], representing a potential public health problem.

Independently of the circumstance that mycotoxins are ubiquitously present in the analysed food or feedstuffs, the found contamination levels were generally low. It is found that only 17% of the AFs tested samples did not comply with the EU maximum permitted level of 5 μg/kg applicable to feedstuffs for dairy animals [144] (Table 1).

The official limit for OTA in cereals intended for direct human consumption in the EU is 5 μg OTA/kg, but for processed cereal products, the limit is lower than 3 µg/kg (Table 2). The official limits and regulations for nephrotoxic mycotoxins in animal feeds in the EU were summarized in several reports [175,176] (Table 1). The accepted limits for OTA in cereals can be found in some FAO reports and usually range from 2 μg/kg (Switzerland) upto 20 μg/kg (Czech Republic) and sometimes up to 50 μg/kg (Uruguay) [176]. Unfortunately, various countries in the world outside the EU have their own limits and regulations for mycotoxins in foods or feeds [177], and internationally recognized limits are still not introduced worldwide. The statutory limits for target mycotoxins in feedstuffs and food products in different countries are usually accepted having in mind the available information, e.g., the available data for the toxicological effect of mycotoxins in different animals or humans, the data on the contamination levels of mycotoxins in food products and feedstuffs, the existing methods of sampling and analysis and the availability of sufficient food supply [176] as well as the existing international trade for each particular country, especially in the case when the export of a particular commodity represents a significant percentage of trade earnings [38]. In this regard, the strict legal measures in developing countries with limited food supplies can lead to food shortages and excessive prices. Therefore, food safety regulations could have significant economic consequences, and the appropriate tradeoffs between health and economic losses should be considered to avoid possible conflict between countries.

Nevertheless, additional efforts are necessary in order to develop internationally recognized regulatory measures for mycotoxins to protect public health issues and simultaneously guarantee fair trade at the international level. Unfortunately, this is not easy to achieve. An easier and more helpful approach would be to introduce some temporary measures, such as guideline limits when there is a significant hazard to public health.

## 4. Compromised Food Safety due to Underestimated Hazard of Multiple Mycotoxin Exposure

Multi-mycotoxin investigations revealed that about 75–100% of the feed or food samples contain more than one mycotoxin at low contamination levels, that additionally could impact animal or human health at low multi-mycotoxin doses [11]. The current regulations for mycotoxins were established considering the toxicological data from studies taking into account only one mycotoxin exposure at a time and do not address the combined toxic effects of several mycotoxins. However, the natural co-occurrence of mycotoxins in food and feed is well-known and explained by the multiple mycotoxin production of each fungus or by simultaneous contamination of food/feed by several fungi. On the other hand, animal diets are usually prepared using multiple grain sources, which contribute significantly to multiple mycotoxin exposure [14]. It is difficult to predict the toxicity of various mycotoxin combinations based on the individual toxicities of each mycotoxin because multiple mycotoxin exposure often has additive, synergistic, or antagonistic toxic effects [108].

Currently, the data on the combined toxic effects of mycotoxins are scarce, and therefore, the health risk from such multiple exposures is not well-known. Grenier and Oswald (2011) reviewed more than 100 cases of mycotoxin interactions and found that most of the same studies reported additive or synergistic interactions of simultaneously co-occurring mycotoxins when addressing the adverse effects on animal performance [107].

In some other studies, Stoev and collaborators [24,25] arrived at a similar conclusion because all analysed feed samples from Bulgaria and South Africa taken from farms experiencing nephropathy problems were found to have multiple mycotoxin contamination, but the contamination levels of OTA were significantly lower than the levels shown to induce nephropathy symptoms. The conclusion was that this nephropathy was mainly caused by the combined synergistic or additive effects of OTA, FB1, and penicillic acid (PA).

In a study from the UK, the screening of maize products intended for animal feed for 22 mycotoxins revealed that all 67 samples were co-contaminated with up to 12 different *Fusarium* mycotoxins [178]. FUMs and DON were found together in 75% of the samples, but moniliformin and ZEA were also frequent co-contaminants. Another study on 330 samples by the same authors revealed that maize was the most affected by co-contamination, and 60% of the studied maize samples were positive for more than one mycotoxin, with AFs and FUMs being the most common mycotoxin combination of about 28% of samples [179].

A similar investigation in Germany revealed the occurrence of 14 different *Fusarium* mycotoxins in 84 German maize samples investigated after the 2006 and 2007 harvests [180]. Likewise, co-occurrence of trichothecenes with ZEA and OTA was seen in 760 Hungarian maize samples [181].

Some other studies on the occurrence of AFs and FUMs in Brazilian maize revealed that 54% of investigated samples were contaminated by both mycotoxins [182]. Such co-contamination with both mycotoxins is particularly concerning because there is some evidence that FB1 synergistically promotes liver tumours initiated by AFB1 [107]. Considering that Brazil is the third-most important producer of maize in the world, the high levels of multiple mycotoxin contamination in Brazilian maize are of international significance.

Another study of feed and feed ingredients in 416 samples from Southern Europe made in 2005 and 2009 revealed that 22% of the compound feed samples contained more than one mycotoxin [183]. In the same study, 23% of samples from Spain and 32% of samples from Italy contained at least two mycotoxins, with trichothecenes, ZEA, and FUMs being the major contaminants and most often co-occurring mycotoxins.

A similar survey in 277 feed samples for fattening pigs in Portugal for OTA, ZEA, and DON revealed that 10% of the samples were found to contain any two-toxin combination, with ZEA and DON co-occurring most often [184].

A similar investigation analyzing 82 samples of sows feed, wheat, and maize from different EU countries in 2008 for the presence of 23 different mycotoxins revealed that 75% of the investigated samples were co-contaminated by two or more mycotoxins [185]. Most of the same samples were co-contaminated with DON and FUMs in combination with some other mycotoxins.

A similar investigation of 50 poultry feed samples in Slovakia investigated for trichothecenes and ZEA revealed that 84% were found to contain two or more mycotoxins. A combination of four mycotoxins, e.g., ZEA, DON, T-2, and HT-2, was found most frequently in 32% of the studied samples [186]. Another study by the same authors on the occurrence of FB1, FB2, and moniliformin in poultry feed from Slovakia revealed that 25% of the samples were co-contaminated with the three mycotoxins [187].

In the study of the diets of dairy cattle in the Netherlands, DON and ZEA were found as co-occurring mycotoxins in 44% of the investigated diets, with silage and compound feed being the major sources of mycotoxin exposure [188].

Some other studies in Europe focused on a single commodity. Among 123 Spanish barley samples from the harvests in 2007 and 2008, which were studied for AFs, ZEA, and OTA, 31% of the samples revealed a co-occurrence of AFB1 and OTA [189]. The co-occurrence of AFB1 and ZEA was found to be 12%, whereas AFB1, OTA, and ZEA co-occurrence was seen in 27% of the samples. In a subsequent study by the same authors on the occurrence of type A and B trichothecenes in the same samples, 43% of them revealed co-contamination with three or more trichothecenes [190]. A further survey combining the data from both investigations showed that 96% of the samples were co-contaminated by three or more mycotoxins [191], with the combinations AFB1, OTA, and DON as well as AFB1, OTA, DON, and ZEA being most frequent and most often observed in 29% and 26% of the samples, respectively.

Presently, it is not quite clear what could be the ultimate effect of prolonged intake of different combinations of mycotoxins as happen in the field. It is supposed that the simultaneous intake of such combinations of mycotoxins, even in low feed concentrations, is possibly a crucial circumstance for developing chronic kidney nephropathy in animals/humans, especially after continuous mycotoxin exposure. This is because the same mycotoxin combinations were found in high (FB1 and PA) or low and moderate concentrations (OTA and CIT) in all feeds originating from farms with nephropathy problems in Bulgaria and South Africa [24,25].

The frequent detection of mycotoxin co-contamination and the evidence on possible additive or synergistic interaction between most co-occurring mycotoxins suggest that guidelines or maximum permitted levels should not only be set for each individual mycotoxin but also for target mycotoxin combinations, found often in feed or food. In order to define such guidelines and rules, more data and further studies are required on the impact of different mycotoxin combinations on various animal species, poultry, or humans. Also, some future studies should be conducted to improve available knowledge on the toxicological effects of co-occurring mycotoxins, which are critical for revising the current guidelines and legislative limits for mycotoxins in feed or food to ensure better health protection for animals and humans. In this regard, possible additive or synergistic interactions with emerging mycotoxins such as moniliformin and beauvericin should also be taken into account [11].

## 5. Concluding Remarks

Obviously, foodborne mycotoxicoses and secondary diseases, which appeared as a response to mycotoxin exposure, are very harmful to animal and human health. Often, the clinical symptoms of foodborne mycotoxicoses are not characteristic, and it is difficult to define their nature. We realise that it is almost impossible to completely prevent animal/human exposure to various mycotoxins, as the same are natural contaminants. Target mycotoxins, e.g., AFB1, OTA, and ZEA, are in increased danger because they actually represent a double safety hazard for animals and humans due to their transmission to humans via animal commodities. A big danger in this regard represents the AFB1 and ZEA content in the milk of lactating cows and either their parent toxins such as AFM1 or αZEA [192] or OTA transmission in eggs or meat [192,193]. Moreover, some of the same mycotoxins, such as OTA, have strong thermal stability at high temperatures or during cooking. The efforts for improving the quality of food or feed should also be aligned with the will of the people to bear any associated increase in the cost of the respective feeds or food products. Obviously, the national programmes for prevention, monitoring, and control of mycotoxin contamination based on the evaluation of the situation in the respective countries are currently unhelpful. In this regard, implementation of well-designed surveillance studies and internationally recognized biomonitoring methods for mycotoxin exposure should be introduced worldwide, in addition to the elucidation of the factors compromising the products’ quality of the commodity system and facilitating the growth of moulds and production of mycotoxins. Also, a well-designed networking system should be elaborated and sustained in other to facilitate the dissemination of available information. The training of appropriate staff should also be conducted either on a regional or international basis. Each opportunity to improve some target multi-mycotoxin biomarker methods should also be investigated, which could improve the collection of reliable and cost-effective blood monitoring data for mycotoxin content. In addition, the cooperation between scientific research institutions, manufacturers, and sellers may help to solve some of the food safety problems. In this regard, it is important to ensure internationally recognized regulations for mycotoxins in food and feed, which should be the final consequence of scientific cooperation between interested countries. The same regulations should be based on recent scientific achievements, including actual food safety, and agree with industry and policymakers in order to ensure the actual protection of the final consumer. However, this is easy to say but incredibly difficult to perform because many circumstances should be taken into account when introducing such regulatory standards. In addition to the recent scientific data, e.g., risk assessment and analytical accuracy in mycotoxin analysis, an additional effort has to be made to elucidate the toxicological effects of a variety of target mycotoxin combinations as occurred in actual field conditions. Moreover, political and economic factors, e.g., the commercial interests of the respective countries, the way to ensure a sufficient food supply for each country, and many other factors should also be taken into account and could play a crucial role in the decision-making process. Nevertheless, such harmonization of mycotoxin regulations and control measures should be undertaken at international levels in order to facilitate food trade between countries and to ensure global food safety based on recent scientific achievements, food safety, and the risk analysis of human health hazards.

Nowadays, the current regulations throughout the world do not address the combined toxic effects of mycotoxins, and only individual mycotoxin effects have been taken into account. It is important to emphasize that the combined effects of mycotoxins should also be extensively investigated because such toxic effects are often synergistic or additive. Some additional permissible limits and international regulations have to be elaborated in regards to combined mycotoxin exposures of animals or humans, especially in the cases when “in vivo” synergistic or additive effects are seen between the respective mycotoxins.

Some additional human or animal studies aimed to explore relationships between the investigated mycotoxins and identified health outcomes (e.g., neural tube defects, idiopathic congestive cardiopathy, or oesophageal cancers described as a consequence of FB1 exposure in humans) are also of crucial importance. In this regard, human or animal exposure can be below the permissible limits for each separate mycotoxin, but the combined toxic effect could be very harmful to animals or humans. Therefore, such a rise in health effects as a consequence of combined mycotoxin exposure should be carefully considered for regulatory purposes.

## Figures and Tables

**Table 1:** Maximum permitted levels or guidance value of target mycotoxins in some feed materials intended for animal feed according to EU legislation and regulation (EC Directive 2002/32/EC, EC Directive 2003/100/EC, EC Recommendations 2006/576/EC and 2013/165/EU) [144,145,146,147].

Mycotoxins	Cereals or Cereal Products	Maximum Level or Guidance Value mg/kg (ppm)
AFB1	-Complete feedingstuffs for cattle, sheep, and goats with exception of the following:	0.02
	✓ Complete feedstuffs for dairy animals	0.005
	✓ Complete feedstuffs for calves and lambs	0.01
	-Complete feedingstuffs for pigs and poultry (except young animals)	0.02
	-Complimentary feedingstuffs for cattle, sheep, and goats (except dairy animals, calves, and lambs)	0.02
OTA	-Cereals and cereal products	0.25
	-Complimentary and complete feedstuffs with exception of the following:	5
	✓ Complimentary and complete feedstuffs for pigs	0.05
	✓ Complimentary and complete feedstuffs for poultry	0.1
DON	-Cereals and cereal products without maize by-products	8
	-Maize by-products	12
	-Complimentary and complete feedstuffs with exception of the following:	5
	✓ Complimentary and complete feedstuffs for pigs	0.9
	✓ Complimentary and complete feedstuffs for calves, lambs, and kids	2
ZEA	-Cereals and cereal products without maize by-products	2
	-Maize by-products	3
	-Complimentary and complete feedstuffs for calves, dairy cattle, sheep (including lamb), and goats (including kids)	0.5
	-Complimentary and complete feedstuffs for piglets, gilts (young sows)	0.1
	-Complete and complimentary feedstuffs for sows and fattening pigs	0.25
Fumonisins FB1 + FB2	Maize and maize by-products	60
	-Complimentary and complete feedstuffs for pigs, horses, rabbits, pets	5
	-Complimentary and complete feedstuffs for poultry, calves, lambs, kids	20
	-Complimentary and complete feedstuffs for adult ruminants and mink	50
	-Complimentary and complete feedstuffs for fish	10
T-2 + HT-2	-Unprocessed barley (e.g., malting barley) and maize	0.2
	-Unprocessed wheat, rye, and other cereals	0.1
	-Unprocessed oats (with husk)	1
	-Oat milling products (husks) for feed and compound feed	2
	-Other cereal products for feed and compound feed	0.5
	-Compound feed, with the exception of feed for cats	0.25
Rye ergot	-All feedingstuffs containing unground cereals	1000

**Table 2:** Maximum permitted levels or guidance value of target mycotoxins in some food products intended for human food according to EU legislation and regulation (EC Recommendation 2013/165/EU and EC Regulation No 2023/915) [147,148] and according to United State Department of Agriculture (USDA-GB2761-2017) [149].

Mycotoxins	Food Products	Maximum Level or Guidance Value (EU) mg/kg (ppm)	Maximum Level or Guidance Value (USA) mg/kg (ppm)
AFB1	-Groundnuts and oilseeds subjected to physical treatment before placing on the market for the final consumer or use as an ingredient in food with exception of that for refined vegetable oil production	0.008	
	
	-Groundnuts and oilseeds used as only ingredient or processed products from groundnuts and oilseeds, placed on the market for the final consumer or used as an ingredient in food with the exception of crude vegetable oils and refined vegetable oils	0.002	
	
	-Tree nuts to be subjected to physical treatment before placing on the market for the final consumer or used as an ingredient in food	0.005	
	
	-Tree nuts used as only ingredient or processed products from tree nuts, placed on the market for the final consumer or used as an ingredient in food	0.002	
	
	-Almonds, pistachios, and apricot kernels subjected to physical treatment before placing on the market for the final consumer or used as an ingredient in food	0.012	
	
	-Almonds, pistachios, and apricot kernels placed on the market for the final consumer or used as an ingredient in food	0.008	
	
	-Hazelnuts and Brazil nuts subjected to physical treatment before placing on the market for the final consumer or used as an ingredient in food	0.008	
	
	-Hazelnuts and Brazil nuts placed on the market for the final consumer or used as an ingredient in food	0.005	
	
	-Dried fruit subjected to physical treatment before placing on the market for the final consumer or used as ingredient in foodstuffs	0.005	
	
	-Dried fruits used as only ingredient or processed products from dried fruits, placed on the market for the final consumer or used as an ingredient in food	0.002	
	
	-All cereals and processed cereal products excluding:	0.002	
	✓ Maize/rice subjected to physical treatment before placing on the market for the final consumer or use as an ingredient in food, and chilies, chili powder, cayenne, white or black peppers, paprika, ginger, turmeric, and nutmeg	0.005	
	
	✓ Processed cereal-based foods and baby foods for infants or children and food for special medical purposes	0.0001	
	-Infant/infant formula/supplementary food, nutritional supplements for pregnant and lactating mothers, formula food for special medical purposes, supplementary food supplements, sports nutrition		0.0005
	-Wheat and its products, barley and its products, other cereals, legumes, and its products, other cooked nuts and seeds (except peanuts), soy sauce, vinegar, brewing sauces		0.005
	-Rice A, brown rice, rice, vegetable oil (except peanut oil and corn oil)		0.01
	-Corn and its products, peanut and its products		0.02
AFM1	-Raw milk, heat-treated milk, and milk-based products	0.00005	
	-Dietary foods for special medical purposes and infant milk	0.000025	
	-Milk and dairy products, infant formula/complementary food, nutritional supplements for pregnant and lactating mothers, formula food for special medical purposes, supplementary food supplements, sports nutrition		0.0005
AFs sum of B1, B2, G1 and G2	-All cereals and processed cereal products excluding:	0.004	
	✓ Maize/rice subjected to physical treatment before placing on the market for the final consumer or use as an ingredient in food, and chilies, chili powder, cayenne, white or black peppers, paprika, ginger, turmeric, and nutmeg	0.01	
	-Dried fruit subjected to physical treatment before placing on the market for the final consumer or used as ingredient in foodstuffs	0.01	
	-Dried fruits used as only ingredient or processed products from dried fruits, placed on the market for the final consumer or used as an ingredient in food	0.004	
	-Groundnuts and oilseeds subjected to physical treatment before placing on the market for the final consumer or use as an ingredient in food, with exception of that for refined vegetable oil production	0.015	
	-Groundnuts and oilseeds used as only ingredient or processed products from groundnuts and oilseeds, placed on the market for the final consumer or used as an ingredient in food with the exception of crude vegetable oils and refined vegetable oils	0.004	
	-Tree nuts to be subjected to physical treatment before placing on the market for the final consumer or used as an ingredient in food	0.01	
	-Tree nuts used as only ingredient or processed products from tree nuts, placed on the market for the final consumer or used as an ingredient in food	0.004	
	-Almonds, pistachios, and apricot kernels subjected to physical treatment before placing on the market for the final consumer or use as an ingredient in food	0.015	
	-Almonds, pistachios, and apricot kernels placed on the market for the final consumer or use as an ingredient in food	0.01	
	-Hazelnuts and Brazil nuts subjected to physical treatment before placing on the market for the final consumer or use as an ingredient in food	0.015	
	-Hazelnuts and Brazil nuts, placed on the market for the final consumer or use as an ingredient in food	0.01	
OTA	-Unprocessed cereals	0.005	
	-Processed cereal products and products derived from unprocessed cereals placed on the market for the final consumer	0.003	
	-Bakery wares, cereal snacks, and breakfast cereals not containing oilseeds, nuts, or dried fruits	0.002	
	-Dried vine fruit (currants, raisins, and sultanas) and dried figs	0.008	
	-Other dried fruits	0.002	
	-Roasted coffee beans, cocoa powder, and ground roasted coffee, excl. soluble instant coffee	0.003	
	-Soluble coffee (instant coffee)	0.005	
	-Wine (incl. wine-based drinks), wine products or cocktails, and grape juice or nectar placed on the market for the final consumer	0.002	
	-Processed cereal-based foods, baby foods for infants/children, and food for special medical purposes intended for infants	0.0005	
	-Dried spices and ginger	0.015	
	-Dried herbs	0.01	
	-Chillies, chili powder, cayenne, paprika,	0.02	
	-Liquorice root, ingredient for herbal infusion	0.02	
	-Liquorice extract for use in food, beverages, and confectionary	0.08	
	-Pistachios subjected to physical treatment before placing on the market for final consumer or use as an ingredient in food	0.010	
	-Pistachios placed on the market for final consumer or used as ingredient in foods	0.005	
	-Sunflower seeds, pumpkin seeds, (water) melon seeds, hempseeds, soybeans	0.005	
	-Non-alcoholic malt beverages	0.003	
	-Wine		0.002
	Nuts and seeds, ground coffee (roasted coffee)		0.005
	-Instant coffee		0.01
PAT	-Fruit juices, fruit nectars, spirit drinks, cider, and other fermented drinks derived from apples or containing apple juice	0.05	
	-Solid apple products placed on the market for the final consumer, incl. compote or apple puree	0.025	
	-Baby foods and apple juice or solid apple products for infants and children, incl. compote or apple puree	0.01	
	-Fruit and its products		0.05
DON	-Unprocessed cereals other than durum wheat, oats, and maize	1.25	
	-Unprocessed durum wheat, oats, and unprocessed maize, with the exception of unprocessed maize intended for wet milling	1.75	
	-Cereals placed on the market for the final consumer, such as flour, semolina, bran, pasta, and germ except for rice products	0.75	
	-Bread and bakery wares, pastries, biscuits, cereal snacks, or breakfast	0.5	
	-Processed cereal-based foods and baby foods for infants and children, except rice products	0.2	
	-Maize flour not placed on the market for the final consumer	1.25	
	-Corn, corn meal (residue, flakes), barley, wheat, cereal, wheat flour		1.0
ZEA	-Unprocessed cereals other than maize	0.1	
	-Unprocessed maize except the maize intended for wet milling	0.35	
	-Cereals placed on the market for the final consumer, such as cereal flour, semolina, bran, and germ, except rice and rice products	0.075	
	-Refined maize oil	0.4	
	-Bread and bakery wares, pastries, biscuits, cereal snacks or breakfast, excluding maize snacks and maize-based breakfast cereals except for rice and rice products	0.05	
	-Maize placed on the market for the final consumer, such as snacks and breakfast cereals	0.1	
	-Processed cereal-based foods and baby foods for infants and children	0.02	
	-Maize flour not placed on the market for the final consumer	0.3	
	-Grain and its products		0.06
Fumonisins FB1 + FB2	-Unprocessed maize, except the maize intended for wet milling	4	
	-Maize and maize-based food placed on the market for the final consumer, excluding:	1	
	✓ Maize-based breakfast cereals and maize-based snacks ✓ Processed maize-based foods or baby foods for infants and children	0.8 0.2	
	-Maize flour not placed on the market for the final consumer	2	
Ergot sclerotia	-Unprocessed cereal grains except for unprocessed rye grains, maize, and rice	200	
	-Unprocessed rye grains	500	
Ergot alkaloids (sum of ergocornine, ergocristine, ergocryptine, ergometrine, ergosine, and ergotamine)	-Milling products of barley, wheat, spelt, and oats (with an ash content lower than 900 mg/100 g dry matter)	0.1	
	-Milling products of barley, wheat, spelt, and oats, incl. barley, wheat, spelt, and oat grains placed on the market for the final consumer (with an ash content equal to or higher than 900 mg/100 g dry matter)	0.15	
	-Rye milling products and rye placed on the market for the final consumer	0.5	
	-Processed cereal-based food for infants and young children	0.02	
T-2 + HT-2	-Cereal grains for direct human consumption:		
	✓ Oats	0.2	
	✓ Maize	0.1	
	✓ Other cereals	0.05	
	-Cereal products for human consumption:		
	✓ Oat bran and flaked oats	0.2	
	✓ Cereal bran except for oat bran, oat milling products other than oat bran and flaked oats, and maize milling products	0.1	
	✓ Other cereal milling products	0.05	
	✓ Breakfast cereals, including formed cereal flakes	0.075	
	✓ Bread, bakery wares, pastries, biscuits, cereal snacks, pasta	0.025	
	✓ Cereal-based foods for infants and young children	0.015	
Citrinin (CIT)	-Food supplements based on rice fermented with red yeast *Monascus purpureus*	0.1	

## Data Availability

Not applicable.
